# Supplementary material for: Anomalous temperature-dependent spin-valley polarization in monolayer WS2
Source: Sci Rep. 2016 Jan 5;6:18885. doi: 10.1038/srep18885 (PMC4700440; doi:10.1038/srep18885)
Supplement: Supplementary Information [file srep18885-s1.pdf]

Supplemental Information

Anomalous temperature-dependent spin-valley polarization in monolayer WS_2

A.T. Hanbicki[1], G. Kioseoglou[2,3], M. Currie[4], C.S. Hellberg[1], K.M. McCreary[1], A.L. Friedman[1], and B.T. Jonker[1]
[1]*Materials Science and Technology Division, Naval Research Laboratory*, Washington, DC 20375
[2]*University of Crete*, Heraklion Crete, 71003, Greece
[3]*Institute of Electronic Structure and Laser (IESL), Foundation for Research and Technology Hellas (FORTH),* Heraklion Crete, 71110, Greece
[4]*Optical Sciences Division, Naval Research Laboratory*, Washington, DC 20375

S1. Sample preparation: different samples used in this study.

Three different samples were used in this study; an optical microscope image of each is presented in Figure S1. The majority of the data presented in this manuscript are from an exfoliated monolayer show in Figure S1a. To confirm the reproducibility of the effects reported in this manuscript, two other samples were also measured. These consisted of a monolayer that we exfoliated from a bulk crystal (Figure S1b) and a sample (Figure S1c) consisting of a large-area single-monolayer, grown at NRL by chemical vapor deposition (CVD). The CVD WS_2 was grown in a quartz tube furnace on SiO_2 (275nm). PTAS seeding particles are used to assist nucleation of the WS_2 on the substrate and are spun on the SiO_2 prior to growth. WO_3 (~1080mg) is placed at the center of the furnace with the SiO_2 directly above it, face-down. Sulfur is in a separate boat upstream in a cooler zone, slightly outside the central heating area. The growth is performed at atmospheric pressure with 100 sccm Ar and 10 sccm H_2 continuously flowing. The heater is ramped to 825˚C and held there for 10 minutes.

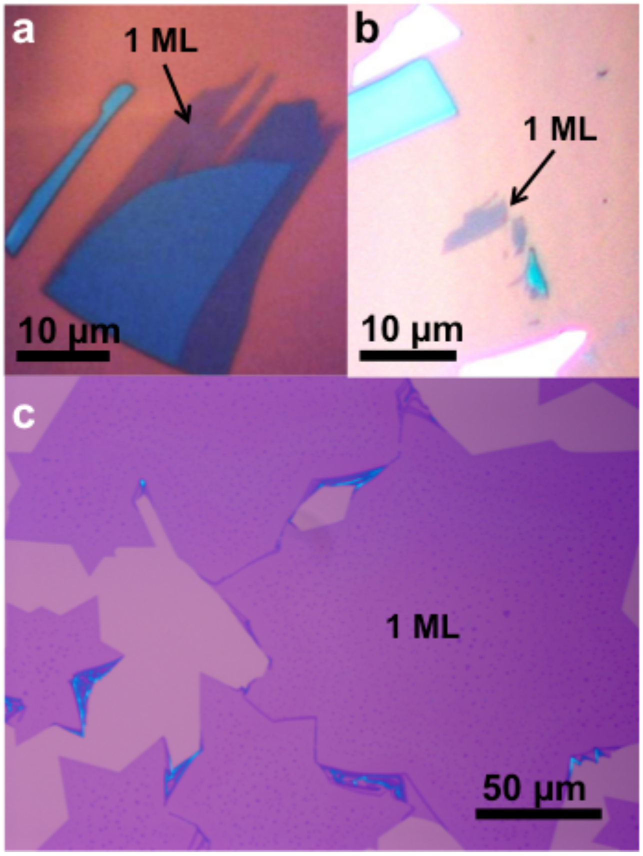

Figure S1. WS_2 samples used in this study. Different exfoliated flakes are shown in (a) and (b). (c) CVD grown WS_2.

S2. Temperature dependent circular polarization.

Figure S2 is a compilation of raw polarization resolved PL spectra of the trion (X–) for all of the excitation energies and temperatures measured on the sample shown in Figure S1a. The incident laser light was polarized $\sigma+$, and the emission was analyzed for $\sigma+$ (red curves) and $\sigma-$ (blue curves). The peak intensities have been normalized to the $\sigma+$ intensity at each temperature and are offset for clarity. The temperature resolved spectra at λ=594 nm and λ=532 nm shown

here are also presented in the main text. When the sample is excited with σ– polarization, identical trends are observed. Figure 3c in the main text is derived from the spectra shown in Figure S2.

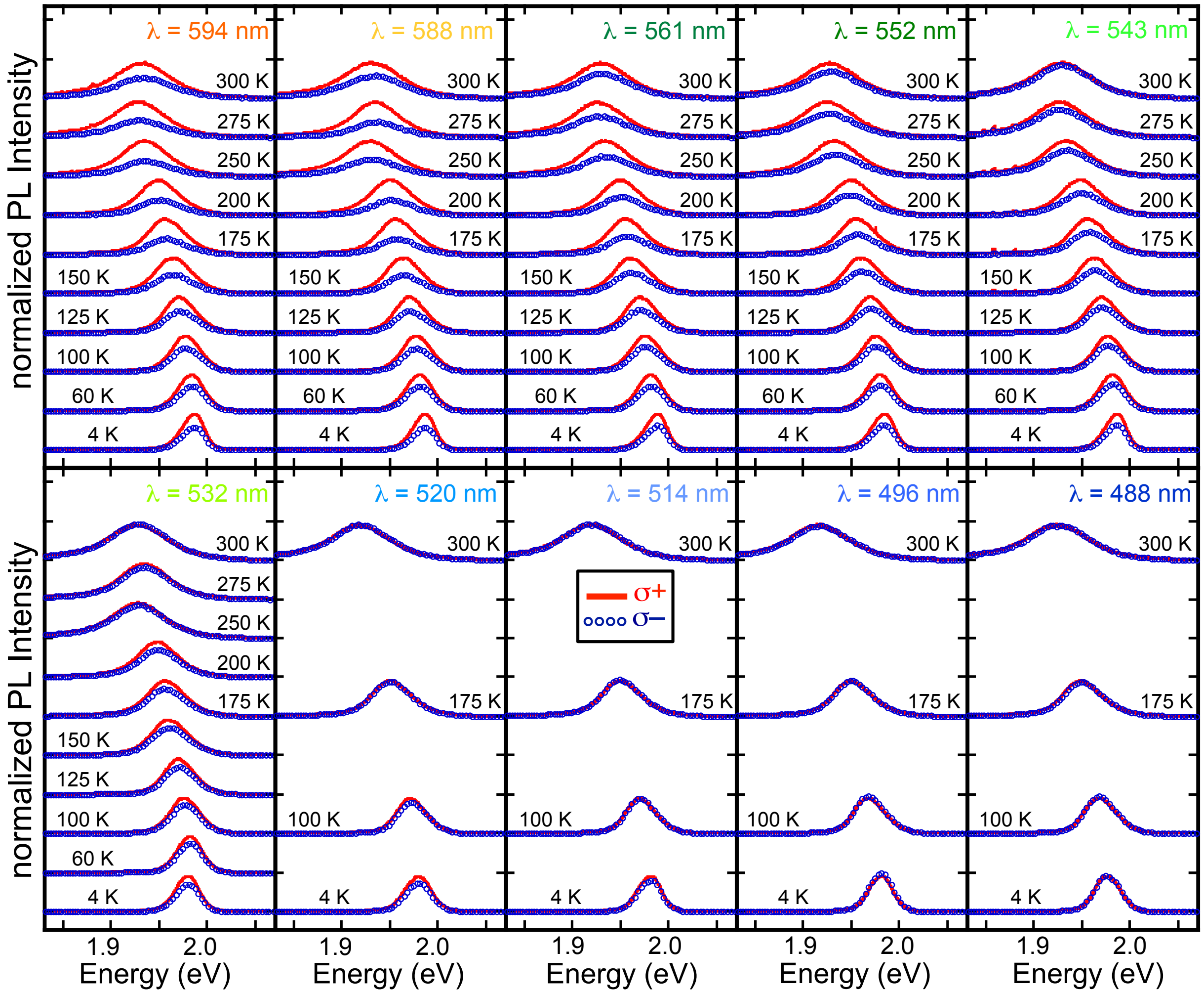

Figure S2. Photoluminescence analyzed for positive (σ+: solid, red trace) and negative (σ–: blue, open circles) helicity as a function of temperature of trion (X–) for 10 different excitation energies (indicated on each panel). At each temperature, the spectra are normalized to the σ+ intensity and offset for clarity.

S3. Rate equation framework.

Within the rate equation framework, the time evolution of population of an exciton in a particular valley, K or K', can be described by the equations:

$$\frac{dN_K}{dt} = g_K - \frac{N_K}{\tau_r} - \frac{N_K - N_{K'}}{\tau_s}, \qquad (1)$$

$$\frac{dN_{K'}}{dt} = g_{K'} - \frac{N_{K'}}{\tau_r} + \frac{N_K - N_{K'}}{\tau_s}. \qquad (2)$$

Here, $g_{K/K'}$ denotes the optical pumping rate of the K or K' valley, τ_r is the radiative recombination time, and τ_s is the spin scattering time, or equivalently in this case the intervalley scattering time. In the main text, rates are used instead of times: the radiative recombination rate $\alpha = 1/\tau_r$, and the spin (or valley) scattering rate is $\beta = 1/\tau_s$. Under steady state conditions $dN_{K/K'}/dt = 0$. We define the polarization as $P = [\, N_K - N_{K'} \,] / [\, N_K + N_{K'} \,]$, which maps to the experimentally measured quantity $P = [\, I(\sigma+) - I(\sigma-) \,] / [\, I(\sigma+) + I(\sigma-) \,]$, where $I(\sigma\pm)$ is the emission intensity analyzed for positive (negative) helicity. The polarization can then solved in terms of the recombination and intervalley scattering time as

$$P = \frac{P_0}{\left(1+2\cdot\frac{\tau_r}{\tau_s}\right)} . \qquad (3)$$

Here, $P_0 = [\, g_K - g_{K'} \,] / [\, g_K + g_{K'} \,]$, is the initial polarization of the system. In the main text instead of these characteristic times, we use the rates α and β. Equations (1) and (2) therefore become

$$\frac{dN_{K/K'}}{dt} = g_{K/K'} - \alpha N_{K/K'} \mp \beta(N_K - N_{K'}). \qquad (4)$$

As the temperature is increased, the recombination becomes a combination of the radiative and non-radiative recombination rates and $\alpha \rightarrow \alpha + A$, where A is the non-radiative recombination rate. The polarization then becomes

$$P = \frac{P_0}{\left(1+2\cdot\frac{\beta}{\alpha+A}\right)}. \quad (5)$$

Figure S3 schematically shows the recombination and scattering processes considered.

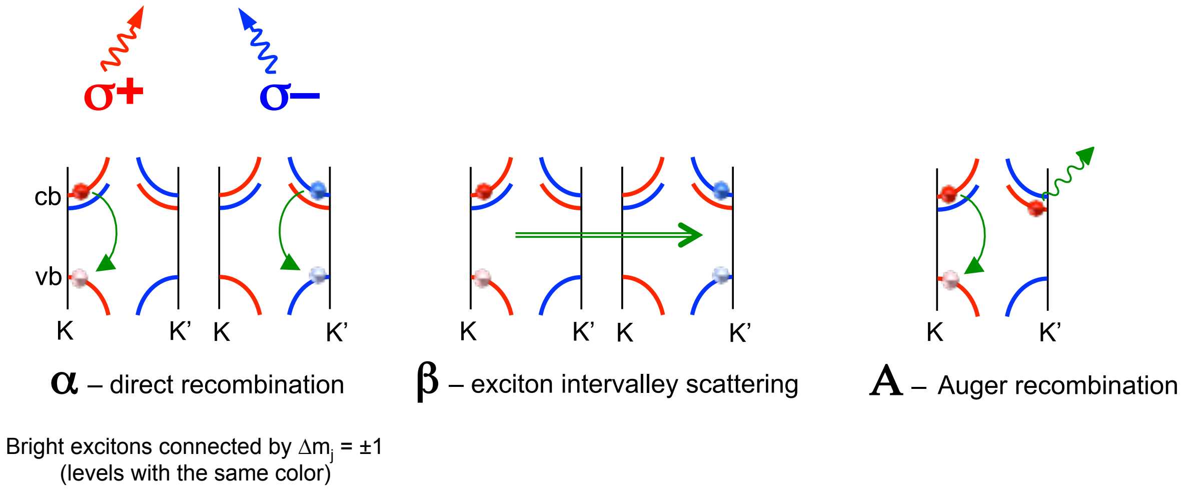

Figure S3. Schematic diagram of the recombination and scattering processes involved in our analysis. These include the direct recombination process of an electron-hole pair, the intervalley scattering of an exciton and the non-radiative, 3-particle Auger process.

S4. Transition from Neutral Exciton to Trion.

In figure 1 and figure 2 of the main text, spectra from the isolated neutral exciton and trion are presented. The system evolves smoothly from neutral exciton to trion, however, and these are only the endpoints. In the intermediate regime, peaks from both the neutral exciton and trion are

visible in the PL and reflectivity spectra. Figure S4 shows the PL (figure S4a) and reflectivity (figure S4b) in this intermediate regime. At low temperature, with a low excitation power, the PL clearly shows both the neutral exciton and the trion. And as is the case for these peaks when they are isolated, the polarization of the neutral exciton is half that of the trion. As stated in the main text, the polarization is defined as $P = [\ I(\sigma+) - I(\sigma-)\ ] / [\ I(\sigma+) + I(\sigma-)\ ]$, where $I(\sigma\pm)$ is the emission intensity analyzed for positive (negative) helicity. The reflectivity at 4 K also shows both peaks and has a separation of 33 meV, the binding energy of the trion.

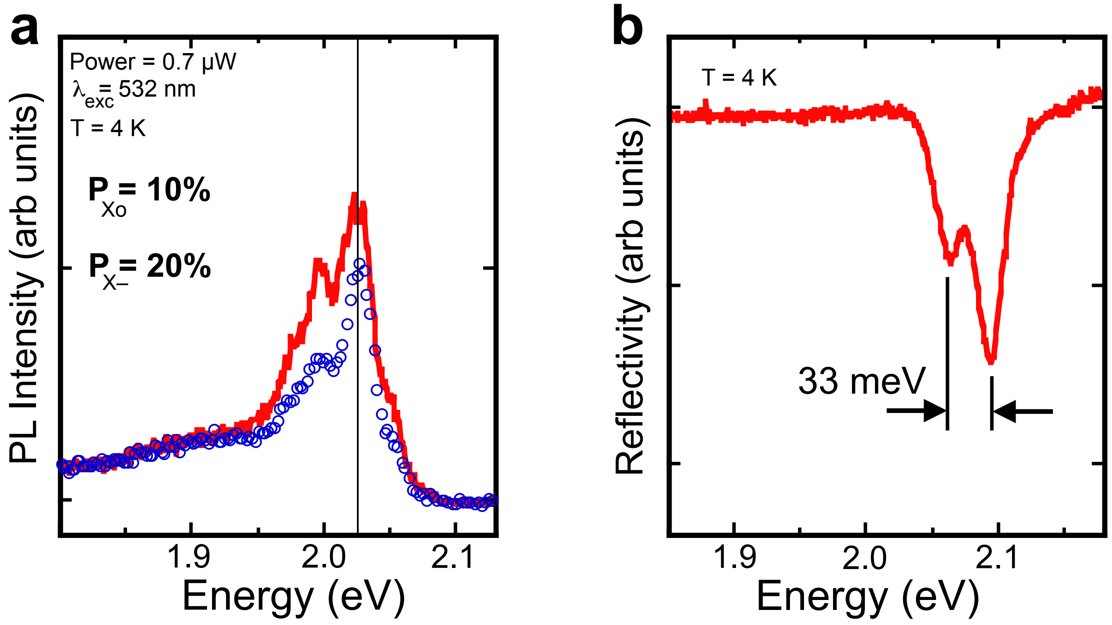

Figure S4. (a) Photoluminescence analyzed for positive (σ+: solid, red trace) and negative (σ–: blue, open circles) helicity at low temperature and power. The trion and neutral exciton are both clearly visible, and with a polarization ratio of 2, as is seen in the spectra where the trion and exciton are isolated. (b) Differential reflectivity at low temperature also showing the coexistent trion and neutral exciton states. The energy difference between these peaks is representative of the trion binding energy.
